# President’s Address

**Published:** 1901-01-15

**Authors:** L. L. Barber

**Affiliations:** Toledo, Ohio


### PRESIDENT'S ADDRESS.

BY L. L. BARBER, D.D.S., TOLEDO, OHIO.

Read before the Ohio State Dental Society, December, 1900.

*Gentlemen of the Ohio State Dental Society*:—We are here to participate in the deliberations of the thirty-fifth annual meeting, and I want to thank this Society for the honor conferred in electing me President.

I do not intend to anticipate the report of any committee, but trust all may be prepared to make such reports as in their judgment seems best. I sincerely hope the Committee on Constitution and By-Laws may report. This work is important and should have had careful consideration.

The Necrology Committee will have a report to make that will bring sorrow to this Society. I refer to the death of our beloved brother, Dr. Charles Welch.

It would seem that dentistry is to be recognized in the army and navy. If I am correctly informed, this bill will surely pass when it comes before Congress. The Surgeon-General advocates the measure, and it practically has received no opposition. If I am not mistaken, the move was resurrected and started afresh in this Society six or seven years ago. Our committee has been very zealous ever since, until all sections have taken it up and pushed it to what is likely to be a final issue. While the bill does not provide as liberally as desired, yet, when the service is once established, its importance must soon be recognized, and ultimately dentistry will get such recognition both as to position and honor in the army and navy as it deserves.

In 1865, at Mt. Vernon, Ohio, a meeting of the Central Ohio Society was held, at which time Dr. J. B. Beaumen, now of Columbus, Ohio, made a motion that a State Dental Society be organized and it was done, and the Central Ohio was absorbed by it.

The objects set forth in its Constitution are as follows:

First. The elevation of the standard of professional education.

Secondly. The advancement and cultivation of dental science and literature.

Thirdly. The protection of the public from the evils of empiricism.

Fourthly. The promotion of the honor, usefulness and interest of the profession and mutual fellowship and good will.

How nearly have we come up to the original objects? I will leave that for you to answer.

Do the members of the Ohio State Dental Society desire any restrictive dental laws in our State? If so, what?

What are the causes of our failures in securing up-to-date dental legislation? I confess I do not know, and mention it that some one may shed some light on the subject.

I want to say a few words upon a subject that to me, at least, is very important.

WHAT IS THE PRESENT STATUS OF DENTAL EDUCATION?

I have no hesitancy in saying that the average dentist of to-day is much better educated than in years past, and it is plain to every observant person, in the profession and out of it, that dental education is having its influence.

Our profession is no longer looked upon as a mere trade, but is ranking well with other honorable professions. But, gentlemen, who are the men who are largely responsible for the good that dental education has accomplished? Are they the rank and file in the profession? Are there not indeed one here and another there who has gone through trials and hardships, which few would care to encounter, to secure high professional attainments, which give us the means for an advanced education?

But while this may have brought dental education to its present status, still there are some points of failure. Are we, in proportion to our ability, doing our duty? Here is the opportunity to analyze our own capacities for the performance of a public duty, which at some time comes to each one of us, that is, the selection and education of a student.

There are very few of us here who have not had more or less experience in this direction, either in or out of colleges. In the light of that experience, is it not a fact that too little discrimination has been used in the selection of the material for the coming dentist?

I can not but feel, after reading the vast number of dental announcements that have come to me this year, that the rapid multiplication of dental schools in the United States is menacing the future of the dental profession.

The aim, in some instances at least, seems to be to bring a large number of students together, without reference to educational or preliminary qualifications, and when they have them, to see how quickly they can get rid of them; for the faster the treadmill works, the more money, and the more money the more effort on the part of these schools to get pupils. Isn't this wrong, and are the schools entirely to blame? They are not!

The demand is for an education that has the greatest commercial value; that which can be the most readily turned into gold. Institutions of learning, of whatever character, are seldom on a level, or in advance of the public demand in the matter of education.

The elevating force must come from without as well as from within. Have we, as dentists, done our part in advising young men to attend only the best schools where they must work in order to attain the degree of perfection necessary to obtain a diploma, or are some of us sending them to institutions whose policy is broad and easy? I fear the latter is true in many cases.

To a great extent the remedy for this evil is in our hands, as individuals. We should recognize the necessity of recommending as pupils only those who possess the natural qualifications for the study and practice of our profession.

The time was when a young man, college or no college, would work out his own salvation, as regards an education, professional or otherwise, and stand or fall by it, and he usually stood.

Conditions are different to-day. Should not we, as dentists, require of a person, who may apply to us for studentship, that he have nearly the following qualifications: A diploma from a good high school having a four years' course, or a diploma from a reputable literary college, or satisfactory evidence that he possesses an equivalent. For well tempered must be the steel and keen the blade in the battle of life. Each man has only what he can seize and hold against unrelenting competition. The brain that thinks and weighs, the eye that questions and discovers, is the one that is in the fight to the end, and not as a mere private, either.

If we, as individuals, should require students to be up to the proper standard, would not the colleges be glad to raise their standards? They know it is too low; that is, many of them do. A few do not care, but they would soon be out of the race.

### ADVANTAGES OF THE DENTAL SOCIETY.

It is just as necessary for a man in dentistry to keep up his knowledge of essential things pertaining to his profession and add thereto, as it is for him to be compelled to have in store an abundance when he begins practice.

In what better way can he put in three or four days every year than in attending the State Dental Association. I believe that any of us would study many times four days to get the knowledge attainable at one of these meetings. This is but one of the good things it accomplishes for us.

It is humiliating to see a man, created in the image of God, sacrificing every pure and noble impulse and the association of friends, with no time to attend dental meetings, or for the cultivation of the higher impulses in the mad rush for gold. Such a one deserves the pity if not the condemnation of every right thinking man. The man who is content to live within his own little shell, or the radius of his own vision, "is little better than the ox that is content to feed on the grass in the meadow, or the worm that crawls in the dust."

It may have occurred to some of those present, who are in the habit of attending dental meetings, that the average dental discussion many times falls far short of what it might be.

It is presumed that papers are read and discussions had thereon to the end that those present may be mutually benefited.

It has occurred to me that the fault, or part of it at least, may be laid at the door of the presiding officer. The best business meetings are those where the most rigid rules are enforced. While our Society is not above criticism in this respect, yet the greatest defect is not here in this body. While a society may err in the letter, it rarely ever is intentionally wrong in carrying out the intent of the laws governing its routine business.

When, after the reading of a paper and the discussion thereon comes up, if the widest latitude is permitted, and extraneous matter is freely admitted; by the time the discussion is well advanced, it bears really no relation whatever to the original subject under discussion.

If it is true that a dental society is for the mutual benefit of its members, then it is the duty of every one connected with it to see that the object is attained in an orderly and effective way.

Any subject under discussion before a dental society is virtually on trial for its accuracy. Those taking part in the discussion are contributors of testimony. The conditions are essentially those upon which cases are tried in legal courts, and it should exclude the possibility of irrelevant matters being introduced, were the same rules adhered to. The introduction of matter, it makes no difference how important it may be of itself, if foreign to the subject under discussion, only tends to confuse the listeners and leaves the impression that after all one is attending an experience meeting.

A closer adherence to the text will admit of a much clearer summing up of the discussion, and a much more definite conclusion may be drawn from the evidence offered.

In conclusion, I want to thank all of the officers and committees, and especially the Executive Committee, for the program it has prepared for this meeting. To those who have never had anything to do with getting up a program for a dental meeting, I want to say—were you to serve but once, I am sure you would thereafter respond more readily when asked to do something for your State Society.

The officers and committees can and do accomplish all they are able, but they can not do everything. It is the duty of every member of this Society to in some way contribute to the sum-total of good accomplished by it. If you do not read a paper or give a clinic, are not one of the officers or on some committee, then make yourself a committee of one, and see that you get at least one more to join the Society, for by helping them and the Society, you can not but benefit yourself.

The influence of this Society for good must depend upon the individual efforts of its members. Our individual resource is soon to be found in the younger men of the profession. The noblest sight this world offers is a young man bent upon making the most of himself. All may not rise equally, yet each one rises according to his deserts. Therefore, our Society will be strong just in proportion as every member develops individual strength, and then brings that strength to the support of the State Society.

---

To Deaden the Mallet Blow.—A rubber hood made of several layers of dam is tied on the plugger. This seems to hasten the recoil, permitting a more rapid use of the mallet and at the same time, by deadening the sound so disagreeable to many patients, apparently softening the blow.—*G. H. Claude, in Cosmos.*

